# TDP-43 Proteinopathy and ALS: Insights into Disease Mechanisms and Therapeutic Targets

**DOI:** 10.1007/s13311-015-0338-x

**Published:** 2015-02-05

**Authors:** Emma L. Scotter, Han-Jou Chen, Christopher E. Shaw

**Affiliations:** 1 Department of Basic and Clinical Neuroscience, Institute of Psychiatry, Psychology and Neuroscience, King’s College London, de Crespigny Park, London, SE5 8AF UK; 2 Department of Pharmacology, University of Auckland, Auckland, New Zealand; 3 Centre for Brain Research, University of Auckland, Auckland, New Zealand

**Keywords:** ALS, FTD, TDP, TARDBP, Proteinopathy, C9ORF72

## Abstract

Therapeutic options for patients with amyotrophic lateral sclerosis (ALS) are currently limited. However, recent studies show that almost all cases of ALS, as well as tau-negative frontotemporal dementia (FTD), share a common neuropathology characterized by the deposition of TAR-DNA binding protein (TDP)-43-positive protein inclusions, offering an attractive target for the design and testing of novel therapeutics. Here we demonstrate how diverse environmental stressors linked to stress granule formation, as well as mutations in genes encoding RNA processing proteins and protein degradation adaptors, initiate ALS pathogenesis via TDP-43. We review the progressive development of TDP-43 proteinopathy from cytoplasmic mislocalization and misfolding through to macroaggregation and the addition of phosphate and ubiquitin moieties. Drawing from cellular and animal studies, we explore the feasibility of therapeutics that act at each point in pathogenesis, from mitigating genetic risk using antisense oligonucleotides to modulating TDP-43 proteinopathy itself using small molecule activators of autophagy, the ubiquitin-proteasome system, or the chaperone network. We present the case that preventing the misfolding of TDP-43 and/or enhancing its clearance represents the most important target for effectively treating ALS and frontotemporal dementia.

**Electronic supplementary material:**

The online version of this article (doi:10.1007/s13311-015-0338-x) contains supplementary material, which is available to authorized users.

## Introduction

Amyotrophic lateral sclerosis (ALS) is the most common adult-onset motor neuron disease, and is characterized by the progressive loss of upper and lower motor neurons from the spinal cord, brain stem, and motor cortex, leading to muscle weakness and eventual respiratory failure. Approximately 5–10 % of ALS cases are familial with the remaining 90 % being sporadic, indicating that both genetic and environmental factors contribute to risk. Despite this diverse etiology of disease, 97 % of patients display a common phenotype in disease-affected tissues, namely the deposition of the TAR-DNA binding protein (TDP)-43 [1, 2]. Deposition of TDP-43 is also the major feature of tau-negative frontotemporal dementia (FTD), which shows clinical overlap with ALS [1, 3]. This convergence of genetic and environmental risk factors upon TDP-43 is hugely informative with regard to general disease mechanisms. Here we explore the pathways linking risk factors to the development of TDP-43 proteinopathy, and linking proteinopathy to the development of disease, with a view to identifying key points for therapeutic intervention.

## TDP-43 and TDP-43 Proteinopathy

### TDP-43 Protein Function

Encoded by *TARDBP*, TDP-43 is a ubiquitously expressed DNA-/RNA-binding protein [4]. TDP-43 contains 2 RNA recognition motifs, a nuclear localization sequence (NLS), a nuclear export signal [5], and a glycine-rich C-terminus that mediates protein–protein interactions [6, 7]. TDP-43 predominantly resides in the nucleus, but is capable of nucleocytoplasmic shuttling [5]. In the nucleus, TDP-43 plays a critical role in regulating RNA splicing, as well as modulating microRNA biogenesis [8, 9]. TDP-43 can regulate the stability of its own mRNA, providing a mechanism for the autoregulation of TDP-43 protein levels [10, 11]. In addition to TDP-43 RNA, TDP-43 regulates the splicing and stability of a large number of other transcripts [10, 12–15], and thus influences diverse cellular processes.

Although mostly nuclear, up to ~30 % of TDP-43 protein can be found in the cytoplasm [16], with nuclear efflux regulated by both activity and stress [17]. TDP-43 is a key component of dendritic and somatodendritic RNA transport granules in neurons [18, 19], and plays an important role in neuronal plasticity by regulating local protein synthesis in dendrites [17]. TDP-43 is also involved in the cytoplasmic stress granule response [20]—the formation of protein complexes that sequester mRNAs redundant for survival [21]—meaning TDP-43 function is particularly important under conditions of cellular stress. Understanding the functions of endogenous TDP-43 is crucial to establishing whether loss of these functions might be key to disease pathogenesis, and to developing effective therapeutics.

### TDP-43 Proteinopathy

TDP-43 protein was identified as a major component of the ubiquitinated neuronal cytoplasmic inclusions deposited in cortical neurons in FTD and in spinal motor neurons in ALS [1]. TDP-43-positive inclusions have subsequently been shown to be common to 97 % of ALS cases [22, 23], whether sporadic or familial. The main exceptions are cases caused by mutations in *SOD1* or *FUS* [24–28]. Neurodegenerative diseases linked to the deposition of TDP-43 are termed “TDP-43 proteinopathies”, and “TDP-43 proteinopathy” also describes the characteristic histopathological transformation of TDP-43 that occurs in disease [29]. This transformation is evidenced by the deposition of full-length and fragmented TDP-43 protein as detergent-resistant, ubiquitinated and hyperphosphorylated aggregates in the cytoplasm, with associated clearing of TDP-43 from the nucleus [1]. The regional spread of TDP-43 proteinopathy from spinal and cortical motor neurons and glia to other cortical regions can be used to stage ALS progression [30], which suggests that some or all of the features of transformed TDP-43 protein are linked to pathogenesis.

However, a key question in ALS research is which of these features of TDP-43 proteinopathy are required for the development of disease and thus represent therapeutic targets. Is ALS pathogenesis linked to the loss of wild-type TDP-43 function through protein misfolding and failure to interact with binding partners, or is it linked to a gain of toxic function of the aforementioned TDP-43 aggregates, which are the hallmark of TDP-43 proteinopathy? A number of studies have examined the roles of TDP-43 gain or loss of function in disease. Overexpression of wild-type TDP-43 recapitulates TDP-43 proteinopathy and disease phenotypes in a range of animal models [31–33], supporting a role for gain of toxic function in disease. Initial studies testing a loss-of-function hypothesis used knock-out of TDP-43 from mice, which resulted in embryonic lethality [34–36]. This demonstrated TDP-43 to play a vital role in early development without necessarily demonstrating that loss of function could be degenerative in adulthood. However, conditional and partial knockout models soon demonstrated that loss of TDP-43 function can, indeed, induce motor neuron defects, a progressive motor phenotype reminiscent of human disease, and even typical TDP-43 proteinopathy [37–39]. Interestingly, either overexpression or knockdown of TDP-43 selectively in glia or muscle also recapitulates ALS-like phenotypes [40, 41]. The emerging picture is that both gain and loss of TDP-43 function may be mechanistic in disease, and, as we will demonstrate, TDP-43 misfolding may link the two. We will demonstrate that therapies that remedy TDP-43 misfolding should be prioritized to best target the spectrum of disease with the fewest assumptions around mechanism.

## Disease Mechanisms in the TDP-43 Proteinopathies

### Disease Mechanisms Upstream of TDP-43 Proteinopathies

We have introduced the concept that TDP-43 is the convergence point for a range of upstream risk factors for ALS. Here we briefly review these genetic and environmental risk factors for the development of ALS, and for the development of TDP proteinopathy, and explore the potential for targeting therapeutics towards these diverse risk factors.

#### Genetic Factors in ALS

**Fig. 1:**
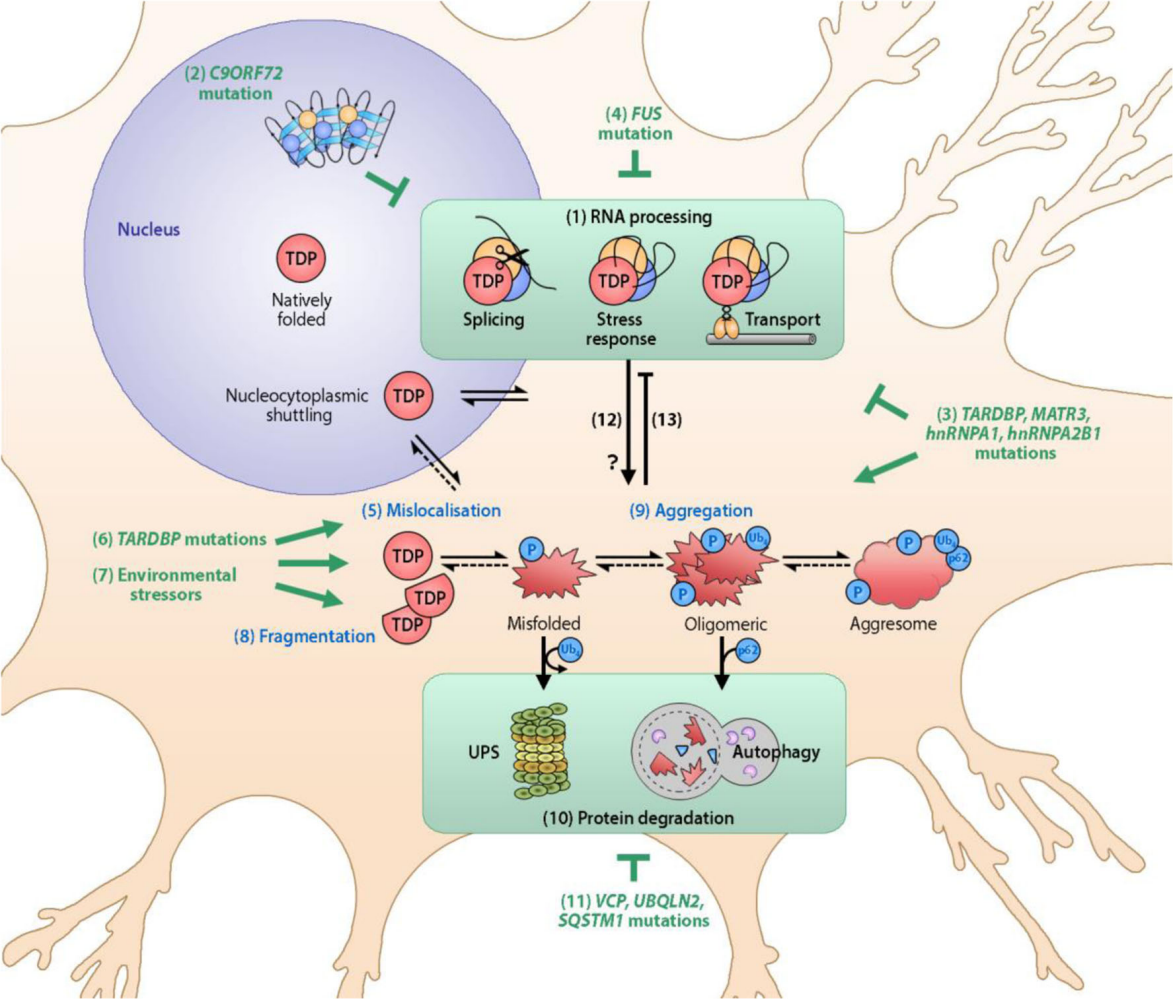
TAR-DNA protein-43 (TDP-43) proteinopathy and its relationship to amyotrophic lateral sclerosis (ALS) pathogenesis. (1) TDP-43 is a DNA- and RNA-binding protein involved in RNA processing. Natively folded TDP-43, shown in the nucleus, regulates RNA splicing. As a nucleocytoplasmic shuttling protein, TDP-43 is also involved in cytoplasmic RNA processing including the stress granule response and RNA transport. (2) *C9ORF72* mutation causes the sequestration of RNA-binding proteins, which impairs RNA processing. *C9ORF72*-mediated ALS also manifests with accumulation and aggregation of TDP-43. (3) *MATR3*, *hnRNPA1* and *hnRNPA2B1* mutations also impair RNA processing and induce TDP-43 proteinopathy, likely through direct binding interactions with TDP-43 which influence its folding and function. (4) *FUS* mutations are thought to cause ALS, independent of TDP-43 proteinopathy, via impaired processing of transcripts that may be common to those targeted by TDP-43. (5) Mislocalization of excess TDP-43 to the cytoplasm can be promoted by (6) *TARDBP* mutations and (7) environmental stressors, both of which also promote (8) TDP-43 fragmentation. (9) Cleaved and mislocalized TDP-43 species are prone to misfolding and aggregation, which is associated with the addition of phosphorylation and ubiquitin chains. (10) The ubiquitin proteasome system (UPS) and autophagy ordinarily serve to maintain TDP-43 homeostasis; however, in ALS these protein degradation systems fail to prevent the accumulation of TDP-43, thus favoring the formation of large protein complexes called aggresomes. (11) Mutations in *VCP*, *UBQLN2*, and *SQSTM1* can impair protein degradation. (12) Aberrant RNA processing, and particularly stress granule formation, may promote the aggregation of TDP-43. (13) Conversely, TDP-43 misfolding and aggregation impairs RNA processing function, and sequesters TDP-43 in a dominant-negative fashion. Strategies that prevent TDP-43 misfolding and/or enhance clearance of pathological TDP-43 have the potential to prevent RNA processing deficits and pathogenesis in the majority of ALS cases. P = phosphorylation; Ub_4_ = tetra-ubiquitin chain

Currently, genetic causes are known for approximately 15 % of all ALS cases; accounting for 11 % of sporadic ALS and 68 % of familial ALS [42]. Many of the ALS-linked genes group together functionally to implicate specific cellular processes in the pathogenesis of ALS (Table 1 and Fig. 1).

**Table 1:** Genetic factors in amyotrophic lateral sclerosis (ALS) and frontotemporal dementia (FTD) implicating RNA processing and protein degradation pathways

	Gene	ALS/FTD	Percentage of cases				TDP-43 deposits	Characteristic features	Refs^*^
			ALS		FTD				
			Sporadic	Familial	Sporadic	Familial			
	Sporadic ALS	ALS	90–95	–	–		+	–	–
RNA processing	*C9ORF72*	Both	4–7	39	6	25	+	DPRs, RNA foci	[177]
	*TARDBP*	Both	1	4	<1		+	–	[42, 159]
	*MATR3*	ALS	1	1	–		+	Matrin 3 elevated/ inclusions	[121]
	*hnRNPA1*	ALS, MSP	<1	2	–		+^†^	hnRNPA1 inclusions^†^	[122]
	*FUS*	Both	1	4	<1		–	FUS inclusions	[42, 159]
Protein degradation	*UBQLN2*	Both	<1	<1	<1		+	UBQLN2 inclusions	[42, 159]
	*VCP*	Both, MSP	1	1	<1		+	Nuclear TDP-43 inclusions	[42, 159]
	*SQSTM1*	ALS, PDB	<1	1	–		+	Increased p62 inclusions	[42]
	*OPTN*	ALS, POAG	<1	<1	–		+	OPTN inclusions	[42]
Other	*SOD1*	ALS	1–2	12	–		–	SOD-1 inclusions	[42]

TDP-43 = TAR-DNA binding protein-43; MSP = multisystem proteinopathy (previously “inclusion body myopathy with frontotemporal dementia Paget’s disease of bone and amyotrophic lateral sclerosis”); PDB = Paget’s disease of bone; POAG = primary open angle glaucoma; DPRs = dipeptide repeats; FUS = fused in sarcoma; UBQLN2 = ubiquilin 2; OPTN = optineurin; SOD-1 = superoxide dismutase-1

*Reference for percentage of cases attributable to the gene

^†^In muscle tissue in MSP. No gene-positive tissue from ALS patients tested

##### TARDBP *mutations*

Mutations in *TARDBP* are a rare cause of ALS [43]. To date, 38 nonsynonymous *TARDBP* mutations have been identified in both familial and sporadic ALS, most clustering in the region encoding the C-terminus, and accounting for approximately 1–2 % of total cases [43–58]. Like wild-type TDP-43 proteinopathy, mutant TDP-43 in *TARDBP*-linked ALS patient tissue is characterized by cytoplasmic accumulation as aggregated and insoluble deposits [44, 55], nuclear clearing in a subset of motor neurons [44], and C-terminal fragmentation [55]. But what is the relationship of mutant TDP-43 proteinopathy to pathogenesis?

Cellular and transgenic animal models indicate that both overt proteinopathy, as well as preproteinopathic changes in TDP-43 may be at play in *TARDBP*-linked disease. Loss of RNA processing and axonal transport function of mutant TDP-43 has been suggested to precede other features of pathology [18, 19, 59], and the failure of mutant TDP-43 to rescue motor neuron defects caused by knockout of endogenous TDP-43 supports the notion that *TARDBP* mutations cause loss of function [18, 39]. However, they also promote C-terminal fragmentation [43, 45, 46, 60], cytoplasmic mislocalization [16, 61, 62], aggregation [18, 63–65], and altered proteostasis [66–69]. These structural abnormalities may therefore underpin both loss of function and proteinopathic transformation of mutant TDP-43. The fact that *TARDBP* mutations cause ALS provides robust evidence that altered TDP-43 structure (i.e., misfolding) and the resultant loss and gain of function is not simply a cellular response to disease but is pathogenic.

We previously demonstrated that a mutant TDP-43 allele (M337V) can be selectively silenced using small interfering RNA [70], and, indeed, mutation-specific therapy may become feasible with the recent demonstration of safety of silencing agents in humans [71]. However, generic TDP-43-based refolding or reduction strategies to be discussed ahead in this review may be more broadly useful, given the number of unique *TARDBP* mutations linked to disease.

##### Protein degradation gene mutations

ALS-linked mutations in *SQSTM1* [72], *VCP* [73], *UBQLN2* [74], and *OPTN* [75] are rare but together implicate impaired protein turnover in TDP-43 proteinopathy and in ALS pathogenesis. They encode p62, valosin-containing protein (VCP), ubiquilin 2, and optineurin, repectively; effectors of the autophagy and/or ubiquitin-proteasome system (UPS) protein degradation pathways [76–80]. TDP-43 proteostasis is normally maintained by the coordinated action of the UPS and authophagy, which is particularly important for clearing TDP-43 oligomers and aggregates [81–87].

Notably, VCP and p62 are required for the formation of “aggresomes” [84, 88, 89], which are large perinuclear inclusions decorated with ubiquitin, ubiquilin, and p62. The TDP-43-positive aggregates that are the hallmark ALS pathology are likely aggresomes [76, 90]. Aggresomes act as a staging center for “aggrephagy”—the removal of misfolded proteins by autophagy [91, 92]—and augmentation of aggrephagy is thus emerging as a potential therapeutic strategy in ALS [90].

But is the removal of TDP-43 a logical approach in ALS linked to global impairment of protein degradation, when TDP-43 is just one of many substrates of these systems? At the high concentrations caused by global impairment in protein degradation, TDP-43, like other neurodegenerative disease proteins [93–95], and RNA-binding proteins (RBPs) [96], is highly prone to aggregation [64]. Thus, TDP-43 may be particularly adversely affected by genetic mutations affecting protein homeostasis. Supporting this idea, the predominant pathology in ALS linked to *VCP* mutation is the nuclear accumulation of TDP-43 rather than VCP protein itself or other degradation substrates [97]. Therefore, it is feasible that the misfolding and accumulation of TDP-43 and not other proteins is the “smoking gun”, and clearance of misfolded TDP-43 might represent a common druggable target.

Certainly, small molecule activators of the UPS or autophagy have been shown to promote TDP-43 clearance and/or mitigate toxicity in models based on TDP-43 overexpression [66, 77, 98, 99]. Autophagy activators may offer selectivity in clearing misfolded TDP-43 [76, 79]; however, given the ability of TDP-43 to autoregulate, even nonselective clearance strategies hold promise for safely restoring TDP-43 proteostasis.

##### RNA processing pathway gene mutations

The identification of disease-linked mutations affecting other RNA-binding proteins suggests an important role for deficits in RNA processing, which is a key TDP-43 function, in disease pathogenesis. ALS-linked mutations in the RBP fused in sacroma (FUS) lead to its mislocalization from the nucleus to the cytoplasm and aggregation [27], but they do not cause TDP-43 proteinopathy [26–28]. However, TDP-43 and FUS do share a subset of RNA targets, which may be part of a disease-relevant pathway [100, 101]. Indeed, RNA targets shared with TDP-43 may also be involved in ALS linked to other RNA processing genes.

The most common ALS-linked mutation is an intronic GGGGCC repeat expansion in *C9ORF72* [102, 103], the pathology of which is characterized by classical TDP-43 inclusions in the motor cortex and spinal cord [102]. *C9ORF72*-linked disease can be distinguished by the additional presence of nuclear foci of repeat-containing RNA [102]. These RNA foci sequester RBPs [104, 105], leading to dysregulation of a large number of transcripts [106, 107]. *C9ORF72* cases also harbor TDP-43-negative inclusions throughout the central nervous system, which are decorated by ubiquitin [102], p62 [14, 108], and/ or ubiquilin 2 [109]. These inclusions contain dipeptide-repeat proteins (DPRs) translated in all 6 frames from repeat-containing RNA [110–112]. Overexpression of DPRs, particularly those that are arginine-rich (polyGR, polyPR) [113, 114], can cause toxicity independently of RNA foci formation [115, 116]. However, the distribution of dipeptide aggregates in ALS/FTD brain and spinal cord, at least for polyGA, shows poor correlation with neurodegeneration [117, 118]. Mapping of arginine-rich DPRs, including preaggregated species, may reveal a role for DPRs in disease, but currently the marker most closely correlated with degeneration remains TDP-43 deposition [118]. Thus, while the reduction of repeat-containing transcript levels or foci formation holds promise in *C9ORF72*-mediated disease [106, 107, 119, 120], TDP-43 misfolding should also be pursued as a potential therapeutic target.

Mutations in the RNA-binding protein genes *MATR3* and *hnRNPA1* are also associated with ALS and TDP-43 proteinopathy [121, 122], potentially through direct binding of the affected RBP to TDP-43 [6, 121]. Taken together with *FUS* and *C9ORF72*, these ALS-linked genes indicate that RNA processing deficits can be a cause of ALS in the presence or absence of TDP-43 proteinopathy, and that rescue of RNA processing defects could be beneficial to patients. As is the case for *C9ORF72*-linked disease, the extent to which RNA processing defects are due to TDP-43 proteinopathy will determine whether TDP-43-based therapeutics are effective in patients.

#### Environmental Stress

For the majority of ALS cases, no genetic mutations have yet been identified to account for disease; therefore, the development of ALS and TDP-43 proteinopathy in susceptible individuals is thought to also involve environmental factors. This idea was first proposed following observations of unexpectedly high disease incidence rates in certain “hotspots”, such as the Kii peninsula of Japan and the island of Guam [36, 123, 124]. While patients in these regions show an increased frequency of ALS-linked genes or genetic modifiers such as *MAPT* [125–128], these only partly account for the observed rates of disease. Proposed environmental agents underlying the high incidence in these populations include dietary neurotoxins such as β-methylamino-L-alanine [129, 130], or mineral deficiencies [75].

There is an association between US military service and increased risk of ALS [131], which may implicate intense physical activity, or exposure to lead, pesticides, or other toxins. Exposure to electromagnetic fields [87, 114], agricultural chemicals [114, 115, 132], head injuries [113, 117], and smoking [116, 119] may also increase susceptibility. Increased incidence of ALS has been reported in professional football players [120, 133, 134]; however, this link has been disputed [135], and risk is not increased for other professional athletes [133]. While no single environmental factor has been unequivocally linked to increased ALS risk, the risk factors discussed can collectively be viewed as cellular stressors, and together implicate cellular stress in disease pathogenesis.

Indeed, cellular studies have drawn links between a diverse range of stressors and the properties of TDP-43 protein. Osmotic stress [136], oxidative stress [20, 137, 138], endoplasmic reticulum stress [66], and heat stress [138] can induce TDP-43 to redistribute from the nucleus to the cytoplasm and incorporate into stress granules. While the regulated aggregation of RBPs and RNA into stress granules is wholly reversible under normal conditions [99, 139], it has been proposed that in conditions of prolonged or repeated neuronal stress, stress granules might act to “seed” the irreversible pathological transformation of TDP-43 [96, 140, 141]. Certainly, TDP-43 within stress granules is detergent resistant and may become post-translationally modified [137, 138, 142], which are defining features of TDP-43 proteinopathy. Also, stress granule markers have been found by several groups to co-localize with TDP-43 aggregates in ALS patient spinal cord [137, 143], although not by others [20, 144]. Together, these findings suggest that myriad and diverse environmental stressors, which normally induce the reversible coalescence of TDP-43 into stress granules, might instead promote irreversible TDP-43 changes and that these are linked to a common pattern of degeneration. A number of antioxidants have been trialed in patients with ALS, unfortunately without success [134], highlighting the need for better understanding of factors which precipitate proteinopathy and disease.

### Disease Mechanisms via TDP-43 Proteinopathy: Toxic Features and Points of Intervention

The vast majority of ALS and FTD cases are of unknown etiology but are linked by TDP-43 proteinopathy, which is defined by cytoplasmic mislocalization, fragmentation, aggregation, and post-translational modification. Here we examine how each of these features of pathological TDP-43 is linked to ALS pathogenesis, and whether preventing these common features might be a valid therapeutic strategy.

#### Cytoplasmic Mislocalization of TDP-43

Mislocalization of TDP-43 in ALS and FTD is evidenced by the deposition of granular, skein-like, and macroaggregated TDP-43 in the cytoplasm, as well as clearing of TDP-43 from the nucleus [1]. Enhanced levels of TDP-43 in the cytoplasm can occur downstream of ALS-linked mutations [16, 39, 60, 145, 146], cellular stress [20, 136–138], or impaired degradation [147]. TDP-43 mislocalization can be induced in model systems by targeted mutation of the NLS; importantly, this sets in motion many pathological features of disease—NLS mutant TDP-43 is cytoplasmic, aggregated, and capable of recruiting wild-type TDP-43 [5]. Interestingly, several studies have shown that increased levels of cytoplasmic TDP-43 are toxic to cells, but that toxicity is independent of inclusion formation at the level of light microscopy [16, 148]. An important question therefore is whether pathogenic TDP-43 in the cytoplasm is natively folded or misfolded.

As a very early pathogenic event cytoplasmic mislocalization is a desirable intervention point, and selectively reducing mislocalized TDP-43 is linked to reduced toxicity *in vivo*. However, studies that demonstrated that link had targeted autophagy or interactions with stress granule components rather than TDP-43 nucleocytoplasmic shuttling per se [99, 141]. Without identifying the unique characteristics of the subset of cytoplasmic TDP-43 that exerts toxicity, developing therapeutics that do not affect TDP-43 undergoing normal nucleocytoplasmic shuttling may be challenging.

#### Fragmentation of TDP-43

Phosphorylated C-terminal fragments (CTFs) of TDP-43 are a major constituent of neuronal protein inclusions in ALS and FTD brains, but less so in spinal cord [1, 149]. Cleavage of TDP-43 is enhanced by C-terminal TDP-43 mutations [45, 43], by cellular stress [150–152], and proteasomal inhibition [124]. Cleavage generates CTFs, which mislocalize to the cytoplasm owing to removal of the NLS, and are aggregation-prone owing to the presence of a prion-like domain [153]. CTFs that correspond in size to those in ALS patient tissue may “seed” the formation of inclusions that are detergent-resistant and ubiquitinated [80], and able to sequester full-length TDP-43 [63, 154–156].

But is fragmentation of TDP-43 a target for therapeutic intervention? There is no current consensus as to whether cleavage enhances or mitigates TDP-43 toxicity [80, 150, 155]. It has been proposed that TDP-43 toxicity requires intact RNA binding capacity [157]; therefore, CTFs may not directly exert toxicity. Cleavage is also not a prerequisite for TDP-43 aggregation [151]; indeed, CTFs expressed at physiological levels require a second “hit” to precipitate misfolding and aggregation [158]. Importantly, cleavage may be required for normal TDP-43 degradation, such that preventing cleavage would favor the accumulation of TDP-43, possibly to greater detriment [124]. Current evidence therefore argues that inhibiting fragmentation of TDP-43 may not be a sound approach for preventing ALS pathogenesis.

#### Misfolding, Aggregation, and Insolubility of TDP-43

The role of TDP-43 aggregation in pathogenesis is one of the most controversial topics in ALS research; thus, preventing aggregation as a therapeutic strategy is equally controversial. Several animal studies have found that mutant TDP-43 causes toxicity in the absence of visible aggregates [59], and preventing visible TDP-43 aggregates from forming failed to reduce toxicity in a cellular model [159]. It should be noted, however, that visible aggregates are only the endpoint of an aggregation pathway that includes a range of TDP-43 species from misfolded monomer to oligomer to mature aggregate [76]. Unfortunately, sensitive methods for detecting misfolded or early aggregated isoforms are seldom employed, so it is difficult to rule out their existence in studies that fail to find aggregates by light microscopy. Certainly, the vast majority of ALS-linked TDP-43 mutations are found in the prion-like C-terminal domain and serve to promote misfolding, which strongly implies that disease pathogenesis is linked, if not to visible inclusions then at least to TDP-43 misfolding [16, 39, 64, 67].

Misfolded mutant TDP-43 shows a reduced ability to transport RNA appropriately [18, 19], constituting a loss of function. Subsequent to misfolding, the formation of oligomers and aggregates of TDP-43 in the cytoplasm may recruit native TDP-43 or its interactors [160]. This constitutes a gain of function, which acts in a dominant-negative fashion, thus essentially also causing loss of function [9, 155]. By restoring proper TDP-43 folding and/or clearing early misfolded TDP-43, we predict that both loss and gain of function toxicity could be abrogated. It is also possible that late TDP-43 aggregates acquire novel toxic functions such as impairment of the proteasome or blockade of axonal transport, but few studies have addressed this. If large aggregates are not a toxic species then strategies that prevent the sequestration of toxic misfolded species into macroaggregates might be detrimental.

There are several intrinsic cellular mechanisms that can act to either prevent or resolve protein misfolding, namely the chaperone system, autophagy, and the UPS. The chaperone system maintains proper protein folding during synthesis and thereafter, or delivers misfolded substrates for degradation [161]. In the case of TDP-43, the chaperone heat shock protein (Hsp)90 enhances solubility (i.e., folding) [162], while HspB8 promotes autophagic clearance of aggregated TDP-43 [163]. Potentiated forms of the disaggregase Hsp104 can mediate TDP-43 refolding [161]. Monomeric misfolded TDP-43 is likely handled by the UPS [76].

Augmentation of chaperones or protein degradation pathways has been protective in several models of ALS and FTD [161, 162]. Because TDP-43 misfolding causes loss of function, as well as providing a substrate for aggregation and gain-of-function/dominant-negative toxicity, we see early misfolding events as one of the most attractive therapeutic targets in ALS with TDP-43 proteinopathy.

#### Phosphorylation and Ubiquitination of TDP-43

In ALS and FTD patient tissue, hyperphosphorylation and ubiquitination are signatures for pathological TDP-43, as they preferentially label TDP-43 that is cleaved, aggregated, and detergent-resistant [164–166]. Phosphorylation appears to precede ubiquitination, and phospho-TDP-43-specific antibodies detect a greater proportion of TDP-43 inclusions in patient tissue [164, 166]. However, the role of post-translational modifications in promoting or preventing TDP-43 toxicity, and the likely therapeutic benefit of targeting them, remains hotly debated.

There are 29 phosphorylation sites on TDP-43 for casein kinase 1 alone [167], the best studied of which are residues 409/410. Casein kinase 2 may also phosphorylate TDP-43 [13, 164]. TDP-43 phosphorylation at 409/410 is not a prerequisite for aggregation [80, 151, 168]. However, when phosphorylation is detected it is almost always associated with misfolding and insolubility of TDP-43. In addition, interventions that modify TDP-43 phosphorylation have been demonstrated to alter its toxicity. Unfortunately, the reported studies conflict over whether TDP-43 phosphorylation mitigates or exacerbates aggregation and toxicity [13, 169–172]. Thus, at present, TDP-43 phosphorylation is of uncertain value as a therapeutic target but can be considered a good marker for gauging the efficacy of therapeutics that aim to modify TDP-43 misfolding.

The influence of ubiquitination on TDP-43 proteinopathy is less well studied. TDP-43 is modified with polyubiquitin chains that are predominantly K48- or K63-linked [7, 76, 173]. However, it is unclear precisely which TDP-43 conformers are ubiquitinated, and what the effects of TDP-43 ubiquitination are. Ubiquitination involving UBE2E ubiquitin ligases causes TDP-43 to shift to the insoluble fraction, but does not promote its degradation [174]. In contrast, Parkin ubiquitination of TDP-43 promotes its cytosolic translocation either with or without an enhancement in degradation [7, 175]. Studies preventing the removal of ubiquitin chains have yielded conflicting answers as to the possible therapeutic utility of targeting TDP-43 ubiquitination. Inhibition of the deubiquitinase USP14 promotes TDP-43 clearance through retention of ubiquitin chains [98]. However, knockdown of the deubiquitinase UBPY exacerbated the toxicity of TDP-43 in *Drosophila*, despite ubiquitin chain retention [174]. The promiscuity of deubiquitinases may limit the therapeutic usefulness of inhibitors in patients; however, augmentation of proteolysis itself, which declines with age [176], remains a potential strategy in ALS, as well as other neurodegenerative diseases.

## Conclusions and Future Perspectives

The contributions of genetic and environmental factors to the etiology of ALS and FTD are complex and interwoven. The increasing accessibility of genotyping and the recent demonstration of safe gene silencing using antisense oligonucleotides may render the targeting of individual gene mutations feasible, but currently this is not the case, except perhaps for *C9ORF72* patients. Similarly, dysregulated RNA processing almost certainly lies at the heart of pathogenesis, but its widespread downstream effects argue against rescue of selected transcripts being useful in patients. Therapeutic strategies would best be directed at a common target proximal to the deficit, which, for most cases, is the misfolding of TDP-43 that is central to its loss of function and gain of function/dominant-negative toxicity. Specifically, enhancers of chaperone-dependent TDP-43 folding, as well as activators of the UPS and autophagy, have shown most promise in model systems. Ongoing refinement of model systems in which to test therapeutics, and recognition of the roles of non-neuronal cell types will be important in bringing these compounds to preclinical and clinical testing. In addition, unbiased large-scale compound screening efforts and the identification of novel causative genes may yield additional insights into disease mechanisms and the role of TDP-43.

## Electronic supplementary material

Below is the link to the electronic supplementary material.

ESM 1 (PDF 1225 kb)
